# Clinical value of ultrasound in adult Wilms’ tumor patient with uremia: A case report and literature review

**DOI:** 10.1097/MD.0000000000036381

**Published:** 2023-12-08

**Authors:** Jing-Jing Zhang, Li-Fei Yang, Yi-Zhen Zhang, Xiao-Hong Xie

**Affiliations:** a Ultrasonic Department, Ningbo Yinzhou No. 2 Hospital, Ningbo, Zhengjiang, China.

**Keywords:** adult Wilms’ tumor, contrast-enhanced ultrasound, ultrasound, uremia

## Abstract

**Rationale:**

Wilms' tumor (WT) is the most common pediatric kidney malignancy and is rarely found in adults. Nonspecific clinical symptoms and imaging features often lead to delayed diagnosis or misdiagnosis of adult WT, resulting in poor clinical outcomes. Ultrasound (US), as an efficient and noninvasive examination method, has been widely used in clinical diagnosis and treatment. Therefore, various US evidence is meaningful to improve understanding of adult WT characteristics in ultrasound.

**Patient concerns:**

A 45-year-old female patient with uremia (regular hemodialysis for 13 years) with painless gross hematuria was diagnosed with a right kidney tumor penetrating to the lung. Preoperatively, B-mode ultrasonography showed an ill-defined hyperechoic mass in the right kidney, which revealed an unclear border, uneven internal echoes, and calcification. Besides, the internal blood flow signal of the tumor was detected. Contrast-enhanced ultrasound (CEUS) showed an uneven hyper-enhancement in the tumor (“fast in and slow out”). Contrast-enhanced computed tomography of the kidney indicated a similar result as the CEUS. Moreover, the chest CT identified multiple pulmonary metastatic nodules.

**Diagnoses:**

An ultrasound-guided percutaneous core needle biopsy of the tumor proceeded to make a definite diagnosis of adult WT (epithelial type).

**Interventions:**

The patient was treated with tislelizumab.

**Outcomes:**

No progress was found to date.

**Lessons:**

We report the first case in which CEUS was performed in an adult WT patient with uremia and multiple pulmonary metastases. The features obtained by the US can help in the diagnosis of adult WT and direct further diagnostic procedures.

## 1. Introduction

Wilms’ tumor (WT), also known as nephroblastoma, is the most frequently diagnosed renal malignant tumor in children, while is rarely found in adults.^[1]^ The prognosis of adult WT patients is relatively poor because of the high malignancy, rapid growth, and early metastasis.^[2,3]^ Due to the low incidence, the lack of specific imaging features, and clinical manifestations, adult WT is always characterized by difficult preoperative diagnosis and a high misdiagnosis rate, which is easily confused with renal cell carcinoma clinically.^[3–6]^

Ultrasound (US) examination is a safe and noninvasive method that has been widely used in renal imaging in general clinical diagnosis and treatment, making it an important initial method for the diagnosis of adult WT after the clinical examination.^[7]^ Therefore, it is valuable and favorable in the clinical diagnosis and treatment fields to understand and master the US features of adult WT.

This report presented the first case of an adult WT patient with uremia and multiple pulmonary metastases. A female uremic patient with painless gross hematuria was diagnosed with adult WT after a series of medical examinations. Furthermore, we summarized its ultrasonographic features by conducting a comprehensive literature review.

## 2. Case presentation

**Figure 1.**
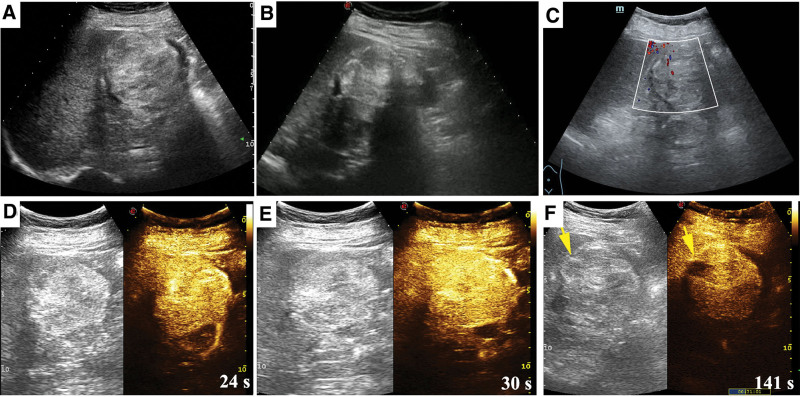
Ultrasonography images of the patient. B-mode ultrasonography revealed an ill-defined hyperechoic mass in the right kidney, with uneven internal echoes (A) and calcification (B). (C) Star-/dot-shaped blood flow detected inside the tumor. Contrast-enhanced ultrasound results showed a “fast in and slow out” pattern in the tumor (D–F) and unenhanced area (F).

**Figure 2.**
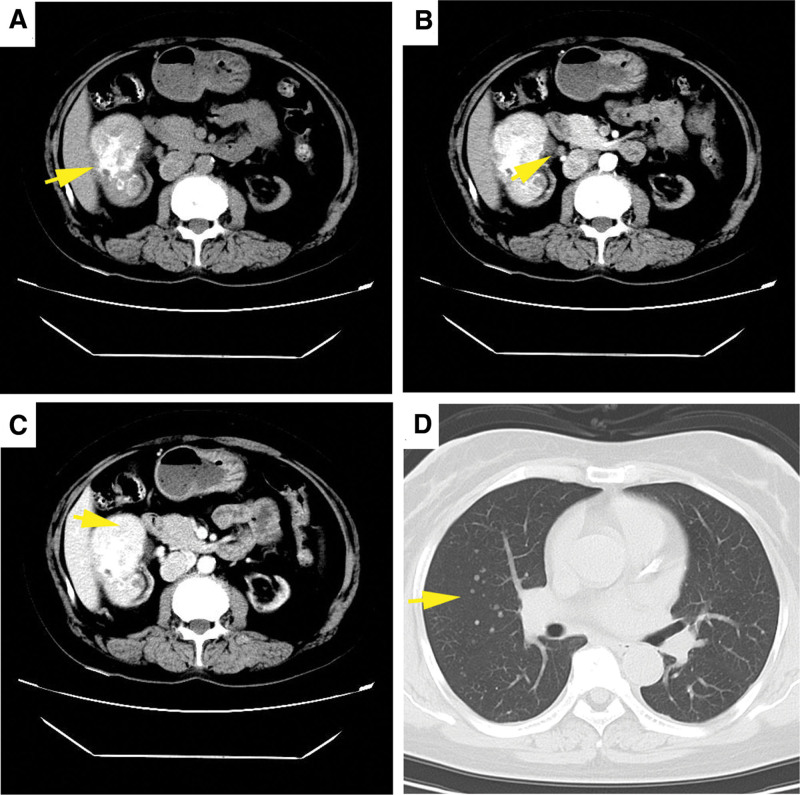
Computed tomography (CT) images of the patient. On the contrast-enhanced CT (CECT), a heterogeneous ill-defined solid-cystic mass was revealed in the right kidney with multiple circular and nodular calcification (A). On the CECT, the parenchyma of the tumor was continuously enhanced, and enlarged lymph node shadows can be seen at the renal hilum, but the cystic component was not enhanced (B and C). The chest CT revealed more new and larger pulmonary nodules (D).

**Figure 3.**
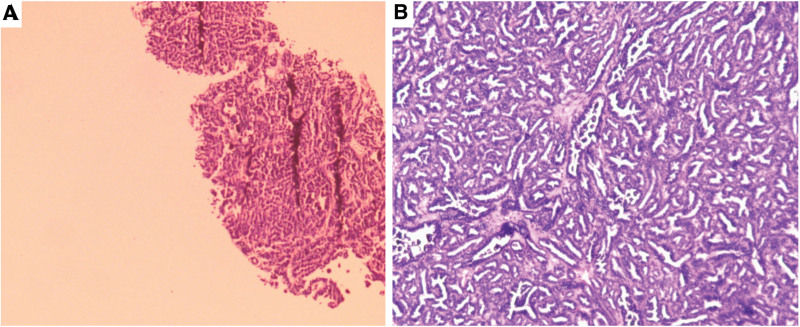
Pathological findings of ultrasound-guided percutaneous core needle biopsy (A) and postoperative mass (B). The mass of the right kidney presents as a small tubular and papillary structure, accompanied by sand bodies and necrosis (hematoxylin and eosin, 40×, 100×).

A 45-year-old uremic female (regular hemodialysis for 13 years) presented to the urology department for painless gross hematuria and took a series of medical examinations. B-mode ultrasonography revealed an ill-defined hyperechoic mass (5.6 cm × 6.3 cm in size) in the right kidney, with uneven internal echoes and calcification (Fig. 1A and B). Star-/dot- shaped blood flow was detected inside the tumor (Fig. 1C). Further, the contrast-enhanced ultrasound (CEUS) results showed a “fast in and slow out” pattern in the tumor (7.3 cm × 6.2 cm in size), and a 1.7 cm × 0.9 cm unenhanced area was found (Fig. 1D–F). The contrast-enhanced computed tomography scan results demonstrated a similar enhancement pattern as CEUS, and there were multiple enlarged retroperitoneal lymph nodes at the right renal hilar level (Fig. 2A–C). Additionally, more new and larger pulmonary nodules were found on the chest computed tomography (CT) compared to those reported 6 months ago (Fig. 2D). The laboratory tests showed that the level of serum ferritin (FERR, 337.5 ng/mL), neuron-specific enolase (NSE, 29.7 ng/mL), and a fragment of cytokeratin 19 (CYFRA 21–1, 5.8 ng/mL) were increased. Due to the non-specificity of the imaging and laboratory examination results, an ultrasound-guided percutaneous core needle biopsy of the tumor proceeded and histopathological examination revealed an adult WT (Fig. 3A). Then, the elective surgery was scheduled and on gross examination, the tumor was about 6 cm large with a 2.5 cm enlarged lymph node near the renal pedicle. Postoperatively, the patient was diagnosed with adult WT (epithelial type) and the tumor metastasis was found in the enlarged lymph node (Fig. 3B). The patient was treated with tislelizumab and no progress was found to date.

## 3. Discussion

WT was named after Dr Max Wilms who first extensively described it in 1899.^[8]^ The incidence of adult WT accounts for <1% of renal tumors, with a prognosis usually poorer as compared to children.^[5,9]^ Besides, due to the extremely low incidence and lacking diagnostic features, it is difficult to distinguish WT from renal masses in adult patients, such as angiomyolipoma, renal cell carcinoma, and eosinophiloma. Therefore, it is essential to enrich the understanding of adult WT characteristics and diagnosis.

The criteria for the diagnosis of adult WT proposed by Kilton et al in 1980 included: a primary renal neoplasm; primitive blastematous spindle or round cell component; formation of abortive or embryonic tubular or glomeruloid structures; no area of tumor diagnostic of renal carcinoma; pictorial confirmation of histology and age >15 years.^[10]^

The presenting symptoms of adult WT include pain, hematuria, and abdominal mass.^[8,11]^ US examination is commonly the first harmless investigation in the diagnosis and treatment procedure due to its obvious advantages of real-time dynamic imaging, cheaper and more widely available than CT or magnetic resonance imaging.^[12]^ Adult WT can penetrate into the renal vein or inferior vena cava,^[13]^ while the US can identify tumor thrombi through multi-dimensional scanning of tumor blood vessels.

Limited cases of adult WT with US evidence have been reported. We analyzed the US characteristics of as many cases found on Pubmed as possible, and the results mainly focused on the following aspects: locations, sizes, morphology, borders, internal echo, blood flow, and CEUS patterns. Generally, adult WT presents as a large ill or well-defined solid rapidly/slowly growing unilateral renal mass with or without variable echogenicity, cystic area, calcification, and internal vascular flow, and the appearances were basically relevant to the components of the tumor (Table 1). Additionally, multilocular and cystic features may indicate a good prognosis. Michikata Hayashida et al reported a 74-year-old man presented with cystic partially differentiated WT and no recurrence occurred in 13 years after the surgical resection.^[14]^ However, due to the rarity of adult WT, more evidence needs to be collected for the diagnosis and prediction. Furthermore, for large solid tumors occurring outside the kidney, WT should not be easily excluded. M A Isaac et al reported a case in which a patient presented a right ovarian multilocular adult WT (19 cm).^[15]^ Serkan Keskin et al reported a 19-year-old man with a 4 cm mass in his lower pole of the right testis by scrotal ultrasonography, which was confirmed to be WT.^[16]^

**Table 1:** Cases of adult WT with ultrasonic findings.

Reference	Age	Gender	Size (cm)	Symptom	Ultrasonic findings of the tumor
I S Morrison^[21]^	17	F	N/A	Right loin pain	(1) In the lower pole of the right kidney; (2) Well circumscribed; (3) Hypoechoic homogenous echogenicity and good sound transmission; (4) No cyst.
	33	M	N/A	Anorexia, weight loss, pain in the left upper limb	(1) In the left kidney; (2) The obliteration of the normal corticomedullary pattern; (3) Solid; (4) Heterogenous pattern with high and low level echo; (5) Ill-defined areas of low level echos.
	21	M	N/A	Right loin, pain, hematuria	(1) In the upper pole of the right kidney; (2) Heterogenous and mainly. hyperechoic in appearance; (3) several echo free areas; (4) Early invasion of the rend vein in the region of the rend hilum.
Y Fukutomi et al^[22]^	49	M	13 × 12 × 15	A painless, ovoid-shaped mass in the left upper quadrant (slowly increasing in size)	(1) In the retroperitoneal region; (2) Uncertain boundary between the mass and the upper pole of left kidney; (3) Well circumscribed; (4) Solid; (5) Several cysts; (6) Echogenicity was almost the same as in the liver parenchyma.
F Kioumehr et al^[23]^	18	F	N/A	A rapidly growing abdominal mass, no weight loss or hematuria	(1) In the lower pole of left kidney; (2) A dilated upper-pole calix; (3) Large, complex, mostiy cystic with septation.
M F Bellin et al^[20]^	39	M	25 × 15 × 15	Abdominal pain, a mass in the left upper quadrant	(1) Its renal origin was suspected (because the mass was contiguous to the lower pole of the left kidney which was displaced downward); (2) Regular margins; (3) Solid; (4) Heterogeneous, including hypo- and hyper-echoic areas.
O Ferrer-Roca et al^[24]^	64	M	12 × 6 × 5	N/A	(1) A malrotated, functioning left kidney; (2) A displaced, cystic right kidney, probably hydronephrotic; (3) Multiple cystic lesions.
N Erdağ et al^[25]^	22	F	6 × 5 × 4	Right flank pain	(1) In cortical layer of the lower pole of the right kidney; (2) Solid; (3) Well-defined; (4) Inhomogeneous.
M A Isaac et al^[15,25]^	21	F	19	Repeated episodes of menorrhagia, a large pelvic mass	(1) In the right ovary; (2) Multilocular; (3) A negative ultrasonographic finding 4 months ago.
Paul T Finger et al^[26]^	37	M	1.5 × 1.3 × 0.49	A primary WT with known disseminated metastasis, orbital pain, metamorphopsia, decreased vision	(1) In choroid of the right eye; (2) Variable internal reflectivity; (3) Irregular tumor surface; (4) Several hyperechoic regions; (5) Retrobulbar edema.
M Pascual Samaniego et al^[27]^	29	F	10	Intermittent renal colic in 2 years	(1) In the upper pole of the right kidney; (2) Cystic (3 cm) initially; (3) Poor Cortical sinus differentiation; (4) Calcification; (5) No urinary tract dilation; Two years later: (1) In the right kidney; (2) Hyperechoic; (3) Solid. (4) Changed renal contour and sinus cavity; (5) Internal punctate calcification; (6) Renal dilation in the upper and middle groups; (7) Internal vascularization.
Hiroshi Masuda et al^[28]^	22	M	N/A	Intermittent right flank Pain in 5 years	(1) In the right kidney; (2) Calcification; (3) Hypovascular; (4) Invading the hepatic lobe.
Navneet Kaur et al^[29]^	25	M	12 × 15	Left flank pain, a progressively increasing lump in left lumbar region	(1) In the lower pole of the left kidney; (2) Hypervascular; (3) The normal renal parenchyma and the hilum pushed superiorly; (4) Normal IVC, left renal vein and right kidney; (5) Invading the left psoas muscle.
Hsi-Lin Hsiao et al^[30]^	66	M	12 × 9 × 6	Painless gross hematuria, poor appetite, body weight loss	(1) In the left kidney.
Nikhil Khattar et al^[31]^	18	F	7 × 5	Recurrent high grade fever, right loin pain and swelling in initially and showed an abdominal mass 1 year later	(1) Right pyonephrosis; One year later (1) In the right kidney; (2) Low echo density; (3) Multiseptate; (4) Intercommunicating spaces and internal echoes (suggestive of pyonephrosis with perinephric extension); (5) Normal left kidney.
Serkan Keskin et al^[16]^	19	M	4	Right testicular swelling	(1) In the lower pole of the right testis; (2) Solid.
James William Ryan et al^[32]^	39	F	22 × 13	Hypertension, a palpable abdominal mass	(1) In the left kidney; (2) poor intrinsic vascularity.
Min-Min Cao et al^[33]^	60	F	6.2 × 5.3 × 3.9	Vaginal bleeding for more than 20 days	(1) Enlargement of the uterine cavity; (2) Cystic; (3) Solid; (4) Heterogeneous; (5) Irregular anechoic fluid areas inside the mass; (6) Normal bilateral adnexa area.
Joseph J Kromka et al^[34]^	50	M	5.5 × 4.6 × 4.3	A painless, palpable mass	(1) In the left testicle; (2) Complex marked heterogeneous echotexture; (3) Cystic and solid components; (4) Prominent vascularity consistent with neoplasm; (5) Normal right testicle.
Krzysztof Ratajczyk et al^[30]^	28	M	7.8 × 6.8 × 8.5	Gross hematuria, right flank pain	(1) In the right kidney; (2) Massive acute deep vein thrombosis in lower limbs and vena cava below renal veins level.
Michikata Hayashida et al^[14]^	74	M	4	Asymptomatic left renal tumor	(1) In the left kidney; (2) Multilocular and cystic; (3) Septa in the middle portion of left kidney.
Suryansh Bajaj et al^[5]^	39	F	12.6	Right sided abdominal pain, fullness	(1) In the inferior pole of the right kidney; (2) Solid; (3) Internal vascular flow; (4) Small cystic spaces; (5) No hydronephrosis; (6) Normal left kidney; (7) No ascites; (8) No hepatosplenomegaly.
Present case	45	F	7.3 × 6.2	Uraemia (regular hemodialysis for 13 years), painless gross hematuria	(1) In the right kidney; (2) Ill-defined; (3) Hyperechoic; (4) Uneven internal echoes; (5) Calcification; (6) Internal blood flow signal; (7) CEUS pattern: uneven hyper-enhancement in the tumor (“fast in slow out,” unenhanced area).

CEUS = contrast-enhanced ultrasound.

This is the first case of an adult WT patient with uremia with CEUS evidence, which provides information valuable for the understanding of adult WT and for better diagnosis. Moreover, the ultrasound-guided percutaneous core needle biopsy is an effective method in the diagnosis with low post-biopsy complications.^[13]^ The US could directly show the extent of the tumor and its position to the adjacent organs; hence, ultrasonography also plays an important role in the follow-up of the treatment results due to the high rates of recurrence and metastasis of adult WT.^[17]^

As for the laboratory results, the increasing level of neuron-specific enolase might indicate the diagnosis of WT,^[18,19]^ which is quite matched with our case. In conclusion, due to the rarity and diversity of adult WT, no conclusive principles are available for the diagnosis and prognosis of this disease. As one of the most routine medical examination methods, the US is of great significance in improving the sensitivity of adult WT diagnosis by combining existing adult WT ultrasonic features with symptoms such as loin pain, asymptomatic gross hematuria, or abdominal masses. Besides, it is critical to consider the results of CT, magnetic resonance imaging, and laboratory examinations and stage the disease in a reasonable way,^[20]^ which is of great help for patients to receive timely treatment and a good prognosis.

## Author contributions

**Conceptualization:** Jing-Jing Zhang.

**Data curation:** Jing-Jing Zhang, Li-Fei Yang, Xiao-Hong Xie.

**Investigation:** Li-Fei Yang, Yi-Zhen Zhang.

**Methodology:** Jing-Jing Zhang, Li-Fei Yang, Yi-Zhen Zhang.

**Supervision:** Yi-Zhen Zhang, Xiao-Hong Xie.

**Writing – original draft:** Jing-Jing Zhang, Li-Fei Yang.

**Writing – review & editing:** Yi-Zhen Zhang, Xiao-Hong Xie.
